# Optimizing acceleration-based ethograms: the use of variable-time versus fixed-time segmentation

**DOI:** 10.1186/2051-3933-2-6

**Published:** 2014-03-28

**Authors:** Roeland A Bom, Willem Bouten, Theunis Piersma, Kees Oosterbeek, Jan A van Gils

**Affiliations:** Department of Marine Ecology, Royal Netherlands Institute for Sea Research (NIOZ), 1790 AB Den Burg, P.O. Box 59, Texel, The Netherlands; Computational Geo-Ecology, Institute for Biodiversity and Ecosystem Dynamics (IBED), University of Amsterdam, P.O. Box 94248, 1090 GE Amsterdam, The Netherlands; Chair in Global Flyway Ecology, Animal Ecology Group, Centre for Ecological and Evolutionary Studies, University of Groningen, PO Box 11103, 9700 CC Groningen, The Netherlands; SOVON Dutch Centre for Field Ornithology, Coastal Ecology Team, 1790 AB Den Burg, Texel, The Netherlands

**Keywords:** Behaviour classification, Change-point model, Crab plover, *Dromas ardeola*, Movement ethogram, Random forest, Supervised classification, Tri-axial acceleration, Video annotation

## Abstract

**Background:**

Animal-borne accelerometers measure body orientation and movement and can thus be used to classify animal behaviour. To univocally and automatically analyse the large volume of data generated, we need classification models. An important step in the process of classification is the segmentation of acceleration data, i.e. the assignment of the boundaries between different behavioural classes in a time series. So far, analysts have worked with fixed-time segments, but this may weaken the strength of the derived classification models because transitions of behaviour do not necessarily coincide with boundaries of the segments. Here we develop random forest automated supervised classification models either built on variable-time segments generated with a so-called ‘change-point model’, or on fixed-time segments, and compare for eight behavioural classes the classification performance. The approach makes use of acceleration data measured in eight free-ranging crab plovers *Dromas ardeola*.

**Results:**

Useful classification was achieved by both the variable-time and fixed-time approach for flying (89% vs. 91%, respectively), walking (88% vs. 87%) and body care (68% vs. 72%). By using the variable-time segment approach, significant gains in classification performance were obtained for inactive behaviours (95% vs. 92%) and for two major foraging activities, i.e. handling (84% vs. 77%) and searching (78% vs. 67%). Attacking a prey and pecking were never accurately classified by either method.

**Conclusion:**

Acceleration-based behavioural classification can be optimized using a variable-time segmentation approach. After implementing variable-time segments to our sample data, we achieved useful levels of classification performance for almost all behavioural classes. This enables behaviour, including motion, to be set in known spatial contexts, and the measurement of behavioural time-budgets of free-living birds with unprecedented coverage and precision. The methods developed here can be easily adopted in other studies, but we emphasize that for each species and set of questions, the presented string of work steps should be run through.

## Background

In trying to achieve a deeper understanding of the functions of, and the mechanisms underlying, animal movement, it helps to know the details of movement in relation to relevant behaviours, especially in well-known field contexts [1]. This requires (1) the technology to measure movements [2, 3] and (2) a classification of behaviours, including different types of movement behaviour [4], a ‘movement ethogram’ as it were. With technology now going far beyond binoculars and notebooks, combinations of animal-borne GPS and tri-axial accelerometer devices present us with a solution to study the whereabouts and behaviour of animals on a precise and near-continuous basis [5]. GPS receivers fix their location, while acceleration data can be used to classify animal behaviour [6].

**Figure 1:**
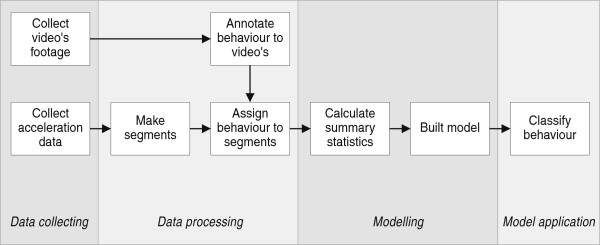
**The eight step protocol for obtaining acceleration-based supervised behavioural classification that was followed during our study.**

Two types of classification approaches can be used to identify behavioural modes in acceleration data. Unsupervised classification algorithms are needed when information on the behaviour is not known at the start of the modelling [7] and after the exercise is done, behaviour is classified based on expert knowledge. Supervised classification algorithms can be built on a labelled dataset [4] and the behaviour classification is a direct outcome of the model. A protocol for obtaining acceleration-based behavioural classification with supervised machine learning algorithms has been outlined previously [4, 8] (summarized with adjustments in Figure 1). The approach has a data collection, a data processing, a modelling, and a model application part. The data collection part consists of acquiring acceleration data and gaining information on the behaviour of the animal on which the accelerometer is mounted. The data processing part consists of dividing the acceleration data into segments, and of assigning a behaviour class to each segment. The modelling part consists of calculating and selecting summary statistics that describe the data and of building the classification model. Finally, in the model application part the model is used to classify behaviour for all the collected data.

A tricky part in this approach is the segmentation. So far, most, if not all studies aiming to obtain acceleration-based behavioural classification [4, 8–15] used fixed-time segments (e.g. of 1 second) as input for classification models. Fixed-time segments may well limit the classification power of the resulting models as they typically can consist of ‘contaminated’ acceleration data that represent two behavioural classes. To overcome this problem the idea of using variable-length segments has been proposed [4] but never fully examinated.

**Figure 2:**
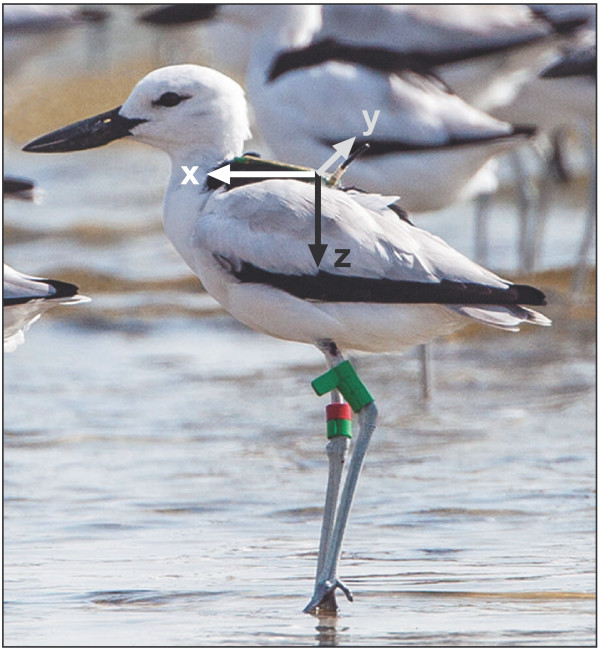
**A crab plover carrying the UvA-BiTS tracker.** The arrows represent the tree-axial acceleration that is measured by the device. Original photo by Jan van de Kam.

In this paper we develop a supervised classification model built on both variable-time and fixed-time segment lengths using acceleration data of free-ranging crab plovers *Dromas ardeola* (Figure 2) moving around and foraging during low tide on the tropical intertidal mudflats of Barr al Hikman in the Sultanate of Oman, and compare the resulting classification performances of both approaches.

## Methods

An eight step protocol for obtaining acceleration-based behavioural classification is summarized in Figure 1. Below we follow the workflow step by step, illustrated with the collected crab plover data and by emphasizing the data segmentation part.

### Data collection

#### Acceleration data

In March 2011, November 2011 and November 2012, respectively 3, 11, and 8 adult crab plovers were fitted with the UvA Bird Tracking System [5] (Figure 2). All birds were caught with mist nets at night. The tracked crab plovers weighed an average of 375 g (SD ± 25 g), mean weight of the trackers and their attachments was 15.1 g (SD ± 0.5 g), so on average the birds had to cope with 4% added mass. The tracking device was solar powered and included a GPS receiver and a tri-axial accelerometer which measured acceleration in three directions: surge (X), sway (Y) and heave (Z). Each direction was measured at 20 Hz. All tracking devices were calibrated to convert the three components of the acceleration data in G-force (1 G = 9.8 m s^-2^). When tags were within reach of the antenna network, both the interval at which the GPS measures as well as the interval and duration at which the accelerometer measures could be changed. During daylight and low tide, trackers were set to measure positions at either 15 or 30 s intervals. Position fixes were always followed by 200 measurements of acceleration (thus, since acceleration is measured at 20 Hz, for a duration of 10 s).

#### Video footage

In November and December 2011 and 2012, during daylight low tides, the intertidal mudflats were searched for tracked birds and whenever a bird was encountered, we filmed it through a 20-60× spotting telescope (Swarovski ATS 80HD) using a Canon VIXIA HG21 camera. We obtained video material on eight birds.

### Data processing

#### Behaviour annotation to videos

We designed an ethogram of eight behaviours (Table 1) and assigned behaviours to acceleration data that could be synchronised with the collected video material using the UvA-BiTS annotation tool (http://staff.science.uva.nl/~bredeweg/pdf/BSc/20102011/DeBakker). The tool will soon be available as a web service (http://www.UvA-BiTS.nl/virtual-lab). We could synchronise 919 bouts of acceleration data of 10 s each with video recordings and in a total of 2,668 instances a class of behaviour was assigned (Table 1).

**Table 1:** **Ethogram of the behavioural classes of crab plovers distinguished on the video recording and the number of assignments per tracked bird**

Behavioural class	Description	# of observations per tracked bird								Total
		#446	#642	#672	#674	#675	#676	#680	#682	
Attack	Fast forwards prey attack, typically followed after a period of waiting	1	3	1	6	0	0	0	26	37
Body care	Cleaning and arranging feathers	21	0	0	18	3	23	0	2	67
Fly	Flying	4	0	0	7	0	0	0	8	19
Handle	Preparing prey for ingestion, usually crabs are stripped on the ground	53	6	1	19	12	3	0	75	169
Inactive	All inactive behaviours, sit, sleep, stand, sit on tarsus, lurk	207	24	56	257	70	77	6	480	1177
Peck	Pecking, similar to attack, but more downwards and slower	17	0	0	11	2	8	0	9	47
Search	The bill is used to sense prey, similar to, but less irregular than handling	56	0	14	31	47	35	0	116	299
Walk	Moving legs forwards	124	16	45	213	60	59	5	331	853

#### Segmentation

As introduced, we make both variable- and fixed-time segments in our acceleration data and subsequently complete the classification procedure (Figure 1) for either approach. Variable-time segments were made using the change-point model framework. This framework provides a method for detecting multiple change points in a sequence, for instance a time series. The models work by evaluating at every possible split point the distribution of a parameter (e.g. mean, variance or both) using a two-sample test statistic [16]. A change point, or in our case a segment boundary, is detected when a set threshold is exceeded. Within the R environment [17], a change-point model is implemented in the ‘cpm’ package [16] that provides the function ‘processStream’. This function uses a test statistics and the parameters ARL_0_ and startup (explained below) to detect sequential changes in a time series. Inspection of the acceleration bouts showed that the *x* signal responds most strongly to a behavioural change by changes in the mean and variance, so here we make segments based on changes in the *x* signal. To do so we used the Generalized Likelihood Ratio (GLR) test statistics which detect both mean and variance changes in a Gaussian sequence. Parameter ARL_0_ corresponds to the average number of observations before a false positive occurs. As we had no expectations, for ARL_0_ we used the values of 500 (the default value), 5,000 and 50,000 (the maximum value allowed) and tested the resulting classification performance for each value (see below). The parameter startup indicates the number of observations after which monitoring begins. The default and minimum value was set at 20, which in our case corresponds with 1 second as acceleration was measured at 20 Hz. As we noticed that crab plovers can change their behaviour within 0.25 seconds, we do not increase the value of startup. Fixed-time segments were made of different lengths, i.e. 0.5, 1, 2 and 3 s.

### Behaviour assignment to segments

**Figure 3:**
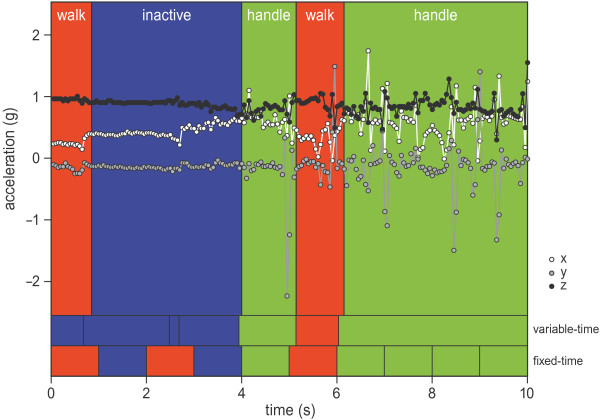
**Example of 10 seconds acceleration data.** The top diagram shows the tri-axial accelerometer data at 20 Hz and in colour the observed behavioural classes. The variable-time row shows the boundaries of the variable-time segments (ARL_0_ = 50,000) and the classified behavioural class. The fixed-time row shows the boundaries of the fixed-time segments (1 sec) and the classified behavioural class. The background colours are unique per behaviour.

Each segment was assigned to a behavioural class (Table 1) that, according to the video annotation, made up most of that segment. Figure 3 shows an example of 10 seconds of acceleration data with variable-time segments (ARL_0_ = 50,000) and fixed-time segments (fixed at 1 second), with both the assigned and classified behaviour.

### Modelling

#### Summary statistics

We calculated summary statistics to characterise the acceleration data within a segment and we used them as features for machine learning. The following were calculated: mean, standard deviation, maximum value, minimum value, skewness, kurtosis, dominant power spectrum, frequency at the dominant power spectrum (Hz), trend, dynamic body acceleration and the overall dynamic body acceleration (ODBA) [4, 8]. Summary statistics were calculated for the *x*, *y* and *z* separately except for the ODBA, which was calculated by taking the sum of the dynamic parts of the three dimensions together. Thus, a total of 31 summary statistics were calculated. The R package ‘moments’ [18] was used to calculate the kurtosis and skewness.

### Model building

The number of behavioural assignments for attack, fly and peck, and to a lesser extent body care, handle and search, were low. We up-sampled the number of observations of attack, fly and peck by a factor six, and of body care, handle and search by a factor two. To this end we used the Synthetic Minority Over-sampling Technique (implemented in the SMOTE function, R package ‘DMwR’), which creates synthetic instances of the minority class using nearest neighbours [19]. For the actual model building part, we applied the random forest supervised algorithm to the selected summary statistics using the R package ‘randomForest’ [20] (default settings used). It was concluded in another study that this method yields the best performance compared to linear discriminant analysis, support vector machines, classification and regression trees and artificial neural networks [4]. Using a resampling procedure, we randomly split the data into two subsamples: 70% of the data was used to train the model and behaviour was classified for the remaining 30% of the data. This classified behaviour was then linked to every single record of acceleration. The classification performance was defined as the number of acceleration records with identical observed and classified behaviour divided by the total number of acceleration records. This procedure was repeated 1,000 times and for each behavioural mode the mean and 95% confidence intervals of the classification performance were calculated. For both approaches we identified settings that yielded the highest classification performance, and used these for further comparisons between the two approaches. For behaviours for which the 95% confidence intervals did not completely overlap, i.e. search, handle and inactive, we compared sample means of the variable-time and the fixed-time approach, using data generated by the resampling procedure. For each behaviour, we calculated the Z-statistic and p-value under the null hypothesis that the means do not differ (i.e. a two-tailed Z-test). The data were logit-transformed to meet the normality assumption.

### Model application

#### Behaviour classification

As an example we show the movement ethogram and the hourly % of time devoted to each classified behaviour of Crab Plover #674 on 20th November 2012, starting 5 hours before, and ending 5 hours after low tide, using the variable-time segmentation approach (ARL_0_ is 50,000).

## Results

**Figure 4:**
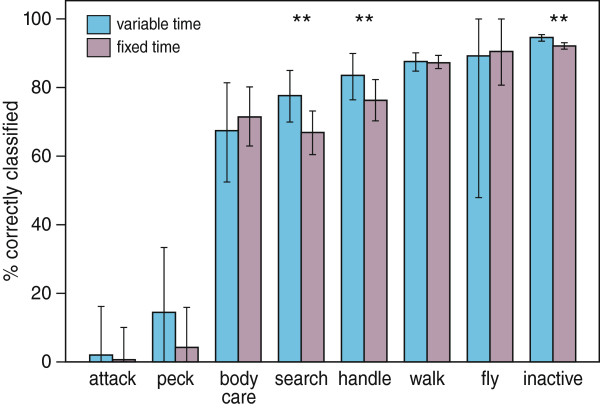
**Results of the variable-time and fixed-time approach with the settings that yielded highest classification performance.** The mean classifications performance and 95% confidence intervals are shown. Significant differences in classification approaches between methods are indicitated on top of the behavioural classes.

Useful classification was achieved by both approaches, but the variable-time segmentation approach considerably outperformed the fixed-time approach for several classes of behaviour (Table 2). The best classification performance for the variable-time segmentation was established when parameter ARL_0_ was set to its maximum value of 50,000. For most behaviours, the best classification performance for the fixed-time approach was obtained when segments were fixed to 1 second. Thus, comparing the variable-time and fixed-time segmentation approach for the settings for which the classification performance was highest (Figure 4), inactive behaviours (95% vs. 92%), flying (89% vs. 91%) a walking (88% vs. 87%), handling (84% vs. 77%), searching (78% vs. 67%) and body care (68% vs. 72%) were reasonably classified with both approaches, and peck (15% vs. 4%) and attack (2% vs. 1%) were never very accurately classified. Compared with the fixed-time segmentation approach, the variable-time segmentation approach yielded a significant higher classification performance for inactive behaviours (Z = 3.12, p < 0.01), handling (Z = 1.50, p < 0.01) and searching (Z = 2.00, p < 0.01).

**Table 2:** **Classification performance (mean percentage and 95% confidence intervals) of the variable-time segmentation approach for different values of ARL**_**0**_ **(upper three rows) and of the fixed-time segmentation approach for different fixed segment lengths (lower four rows)**

Segmentation	ARL_0_	Fixed length (s)	Attack (%)	Body care (%)	Fly (%)	Handle (%)	Inactive (%)	Peck (%)	Search (%)	Walk (%)
Variable-time	500		5.0 (0–24.1)	65.9 (50.3-78.0)	89.8 (55.6-100)	77.6 (70.5-84.4)	94.5 (93.5-95.4)	13.5 (0–28.6)	74.5 (67.0-81.6)	87.6 (85.2-89.7)
	5,000		2.8 (0–17.5)	64.4 (47.3-77.7)	87.8 (52.7-100)	80.3 (73.0-86.7)	94.8 (93.8-95.7)	17.2 (0–33.5)	77.1 (69.2-84.8)	87.8 (84.9-90.2)
	50,000		2.2 (0–16.2)	67.6 (52.4-81.4)	89.4 (47.7-100)	83.7 (76.3-90.1)	94.7 (93.6-95.6)	14.5 (0–33.6)	77.7 (69.8-85.3)	87.8 (84.8-90.3)
Fixed-time		0.5	7.3 (0–21.9)	64.6 (57.2-71.3)	87.6 (78.3-95.7)	70.4 (65.6-75.3)	93.9 (93.0-94.8)	0.4 (0–5.2)	66.3 (60.9-71.8)	88.5 (86.7-90.1)
		1	0.7 (0–10.2)	71.6 (62.8-80.3)	90.8 (80.8-100)	76.5 (70.1-82.4)	92.2 (91.0-93.3)	4.1 (0–16.0)	67.0 (60.4-73.2)	87.4 (85.4-89.5)
		2		62.7 (49.6-75.6)	90.8 (77.9-100)	76.6 (68.5-84.2)	88.2 (86.3-89.8)		61.8 (53.2-68.9)	82.5 (79.6-85.5)
		3		50.4 (35.0-66.0)	95.0 (83.7-100)	73.0 (62.9-81.8)	85.0 (82.8-87.3)		46.9 (37.5-56.5)	80.8 (76.6-84.5)

**Figure 5:**
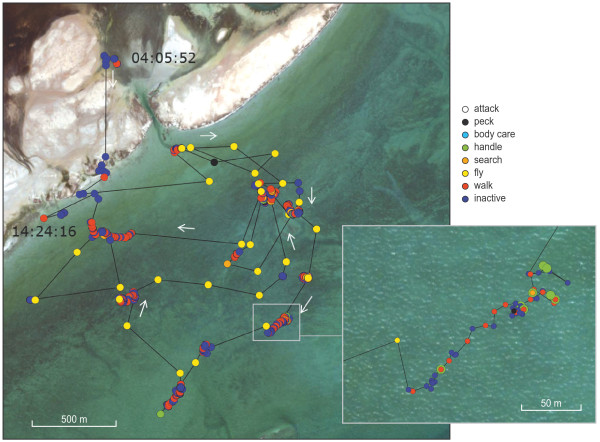
**Movements of crab plover #674 during a single low tide on 20 November 2012.** The time between points is, in general, 30 seconds during low water and 10 minutes during high water. Lines connect subsequent measured positions. After each measured position, acceleration was measured during 10 seconds. Acceleration-based behaviour classification was done using the variable-time segmentation approach. In the enlargement, the point size of handling is slightly larger for visual reasons. The hourly time budget for this example is shown in Figure 6.

**Figure 6:**
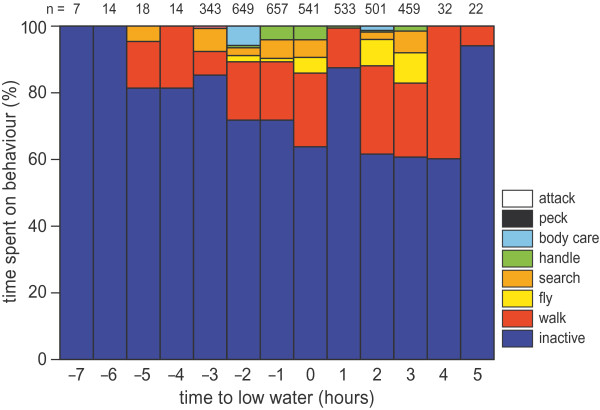
**Hourly time budget constructed from accelerometer data for crab plover #674 during a single low tide on 20 November 2012, using the variable-time segmentation approach.** N-values refer to the number of segments. Behaviours are ranked from least to most occurring.

Figure 5 shows the ‘movement ethogram’ of crab plover #674 during a single tide on 20 November 2012. This example starts around 04 o’clock when the crab plover is inactive at its shoreline roost. With the ebbing tide, the bird goes to the mudflat where it moves between and within distinct areas, which we here call patches. Between patches the bird travels by flight. Within patches the crab plover mainly walks and is inactive and occasionally is searching for, or handling a prey. The example ends in the early afternoon when the water has reached the beach and the crab plover starts to be more inactive. The time budget in Figure 6 suggests that off the mudflats crab plovers are mainly inactive and sometimes walk.

## Discussion

### Variable-time segmentation for acceleration based behaviour classification

We explored the use of variable-time segments and fixed-time segments for developing acceleration-based behavioural classification. By implementing variable-time segments to our data, very useful levels of classification performance were achieved for almost all behavioural classes, levels that were not always achieved by using fixed-time segments. Especially, the implementation of variable-time segments enabled us to satisfactorily raise the classification performance of two behaviours that may look similar in nature; i.e. handle and search (Table 1). These are behavioural classes we are particularly interested in from an ecological point of view (see below).

Given our results we think that other studies developing acceleration-based behavioural classification models will likely raise their classification performance when using the variable-time segmentation approach. Yet, we also realise that the extent to which this is true will depend on the kind of acceleration data that is available, on the studied species and on the aim of the study. The variable-time segmentation approach will be of limited use when few acceleration records are available (i.e. < 20), or impossible when the acceleration data are already summarized by the manufacturer [21]. Also, studies on animals that have short sequences of vigorous behaviours (certainly true for crab plovers that are typical ambush predators which rapidly attack their prey after relatively long motionless waiting bouts) will benefit more from variable-time segmentation than studies that use data collected on animals that have long-lasting behaviours that are slow by nature, e.g. cows [12]. Similarly, variable-time segmentation is probably not needed when the aim of the study is to classify only obviously distinct behaviours such as inactive versus active.

### Application

The present calibration study enables us to study spatial distributions in relation to the behaviour of free-living crab plovers during their non-breeding season at unseasonable hours and inaccessible sites with exceptional coverage and precision. For instance, we can emphasize when and where crab plovers are inactive, when they are searching for prey and how often they handle prey, day and night (crab plover forage during low tide, day and night), we can study which prey is selected (the distribution of the crab plover prey is spatially segregated (R.A. Bom, unpublished data)), predict the sizes of prey ingested (handling time in crab plovers is log-linear related with the size of the crab that is ingested (R.A. Bom, unpublished data)), estimate the (relative) energy expenditure of different behavioural classes [22] and, since crab plovers fly between foraging sites (Figure 5) and since accelerometers indirectly measure wing-beat frequency while flying, we could potentially measure the increase of body mass before and after foraging [23]. As crab plovers travel between patches by flight we can also identify patch giving-up decisions [24]. Together with field experiments measuring digestive constraints of crab plovers (R.A. Bom unpublished data), we can analyse if, where and when prey intake of crab plovers is constrained by searching, handling and or digestive breaks. Furthermore, search and handling are the key input behaviours to the quantification of the relationship between predator intake and prey densities, the ‘functional response’ [25], which is the first step in mechanistically understanding the spatial distribution of (foraging) animals [26, 27].

## Conclusions

Techniques to analyse acceleration data are beginning to appear in the ecological literature. A growing number of studies has developed supervised classification algorithms that satisfyingly classify behavioural modes of the studied individuals [4, 8–16], for other individuals of the same species [28] and even classify behaviour beyond the species level [29]. Outperforming the resolution of more traditional telemetry e.g. [30, 31], especially when accelerometers are combined with GPS sensors, the new methods have great potential for movement ecology. Nevertheless, acceleration-based behavioural classifications have not been successful to classify all behavioural categories accurately (e.g. [8, 15], our study). In our case, the low classification performance for some behaviours was probably due to a low sample size, but also due to the short-lasting nature of the behaviour (this is true for both attack and peck) and of the acceleration-signal being very similar to other behaviours. Thus, future studies are challenged to come up with techniques that can identify such hard-to-distinguish behaviours. These techniques may involve optimization of either of the essential steps in the presented workflow (Figure 1). Our contribution to optimize acceleration-based behavioural classification was to include a variable-time segmentation of the acceleration data. The inclusion of the variable-time segmentation enabled us develop a model that could classify several behavioural modes in crab plovers at satisfying levels. By combining the behaviour classifications with simultaneously measured location data, we were able to make ‘movement ethograms’ on a near-continuous basis with coverage and precision that are unprecedented in the field of movement ecology.

## Availability of supporting data

Supporting data are available upon request to corresponding author.
